# Behaviors in Advance Care Planning and ACtions Survey (BACPACS): development and validation part 1

**DOI:** 10.1186/s12904-017-0236-6

**Published:** 2017-11-22

**Authors:** Aliya Kassam, Maureen L. Douglas, Jessica Simon, Shannon Cunningham, Konrad Fassbender, Marta Shaw, Sara N. Davison

**Affiliations:** 1 Department of Community Health Sciences, Cumming School of Medicine, University of Calgary, 3330 Hospital Drive NW, Calgary, AB T2N 4N1 Canada; 2 Advance Care Planning CRIO Program, Division of Oncology, University of Alberta, Edmonton, AB Canada; 3 Department of Oncology, Community Health Sciences and Medicine, Cumming School of Medicine, University of Calgary, Calgary, AB Canada; 4 Alberta Innovates, Performance Management and Evaluation, Edmonton, AB Canada; 5 Covenant Health Palliative Institute, Edmonton, AB Canada; 6 Community Health Sciences, University of Calgary, Calgary, AB Canada; 7 Department of Medicine, University of Alberta, Edmonton, AB Canada

## Abstract

**Background:**

Although advance care planning (ACP) is fairly well understood, significant barriers to patient participation remain. As a result, tools to assess patient behaviour are required. The objective of this study was to improve the measurement of patient engagement in ACP by detecting existing survey design issues and establishing content and response process validity for a new survey entitled Behaviours in Advance Care Planning and ACtions Survey (BACPACS).

**Methods:**

We based our new tool on that of an existing ACP engagement survey. Initial item reduction was carried out using behavior change theories by content and design experts to help reduce response burden and clarify questions. Thirty-two patients with chronic diseases (cancer, heart failure or renal failure) were recruited for the think aloud cognitive interviewing with the new, shortened survey evaluating patient engagement with ACP. Of these, *n* = 27 had data eligible for analysis (*n* = 8 in round 1 and *n* = 19 in rounds 2 and 3). Interviews were audio-recorded and analyzed using the constant comparison method. Three reviewers independently listened to the interviews, summarized findings and discussed discrepancies until consensus was achieved.

**Results:**

Item reduction from key content expert review and conversation analysis helped decrease number of items from 116 in the original ACP Engagement Survey to 24–38 in the new BACPACS depending on branching of responses. For the think aloud study, three rounds of interviews were needed until saturation for patient clarity was achieved. The understanding of ACP as a construct, survey response options, instructions and terminology pertaining to patient engagement in ACP warranted further clarification.

**Conclusions:**

Conversation analysis, content expert review and think aloud cognitive interviewing were useful in refining the new survey instrument entitled BACPACS. We found evidence for both content and response process validity for this new tool.

**Electronic supplementary material:**

The online version of this article (10.1186/s12904-017-0236-6) contains supplementary material, which is available to authorized users.

## Background

Advance care planning (ACP), “is a process that supports adults at any age or stage of health in understanding and sharing their personal values, life goals, and preferences regarding future medical care. The goal of advance care planning is to help ensure that people receive medical care that is consistent with their values, goals and preferences during serious and chronic illness.” [1].

Advanced care planning includes a range of behaviors such as conversations with physicians and family; choosing a surrogate decision maker (or “agent”); clarifying wishes for future healthcare and legally documenting these decisions (e.g. in an advance directive). Patient engagement refers to patients being critical stakeholders in their healthcare and decision making; having a role in improving the quality and safety of health care interventions and service delivery; and promoting personal health experiences [2]. Patients with advanced illness however, may not be well informed about their prognoses or their available care options and subsequently, engagement in ACP behaviors may be low [3, 4]. Multiple barriers to patient participation in ACP have been described [5].

Ideally, ACP interventions should be tailored to the readiness of individuals to engage in the relevant behaviors. Developing tools to assess changes in patient behavior, or even readiness to engage in a series of behaviors, will likely be central for evaluating ACP patient resources and interventions (e.g. decision aids such as videos and guides,). Such tools are also needed to help health systems evaluate the impact of implementation of ACP programs or quality improvement initiatives [6]. Behavior change theories provide a necessary framework for the development and analysis of these tools/measures.

The purpose of this study was to 1) develop a short research tool that is acceptable to chronically ill patients to measure ACP patient engagement in routine clinical encounters; and 2) provide evidence for content and response process validity for the new tool: The Behaviors in Advance Care Planning and ACtions Survey (BACPACS). We selected the 116-item ACP Engagement Survey [7] by Sudore et al., as the best starting point for the development of the new ACP engagement measure, the BACPACS. This is because Sudore’s (2013) PREPARE survey builds further on knowledge of behavior change, adding an important aspect of behavior change, namely self-efficacy. Additional file 1 outlines the theoretical framework, specifically the trans-theoretical model (TTM) of health behavior change [8, 9] and rationale for the decision to start with the ACP engagement survey. We also provide a comparison of existing tools in Additional file 1.

## Methods

### Ethics

Study approval was obtained from the University of Alberta Health Research Ethics Board (UA HREB 00047324). Furthermore, given the sensitive nature of this topic several safeguards were put in place during the research process. First the research assistant pointed out to the participant during consent and again at the end of the interview “that none of the information *communicated* during the study would be shared with their care team, and that the participant is responsible for communicating directly with the team about anything that is important to them.”

Second, the research assistant also explicitly stated to the patient: “Remember, the information you provide is for research purposes only and will remain strictly confidential. The healthcare workers directly involved in your care will not see your responses to these questions – if you wish them to have the information, please bring it to their attention yourself.”

Last, our research training manual for research assistants clearly stated “[At the end of the interview] remind the participants again that nothing communicated during the survey will be shared with their health care team. If the respondent requests more information regarding ACP please ask them to speak to their health care team.”

### Methodology

In developing and providing evidence for content validity of BACPACS, we used the framework by Samuel Messick [10] adopted by the American Educational Research Association (AERA), American Psychological Association (APA), and the National Council on Measurement in Education (NCME) as a field standard [10, 11]. In this framework, all forms of validity are considered to be construct validity, and evidence for the presence of validity is collected from five different sources: content, response process, internal structure, relations with other variables, and consequences [12]. This study sought evidence for content and response process validity evidence.

Content validity ensures that the tool’s content is representative of the intended measurement construct(s). In keeping with Messick’s framework, development of BACPACS and establishment of evidence for content validity incorporated: 1) consensus on the theoretical framework and constructs to conceptualize ACP behaviors; 2) formulation based on prior instruments; and 3) expert review. Response process evidence is an evaluation of how well the participants’ actions relate with the intended measurement construct. This includes: quality control and the analysis of the respondents’ thoughts and/or actions during completion of the survey tool [11]. All these components were integrated into the methodology.

**Fig. 1:**
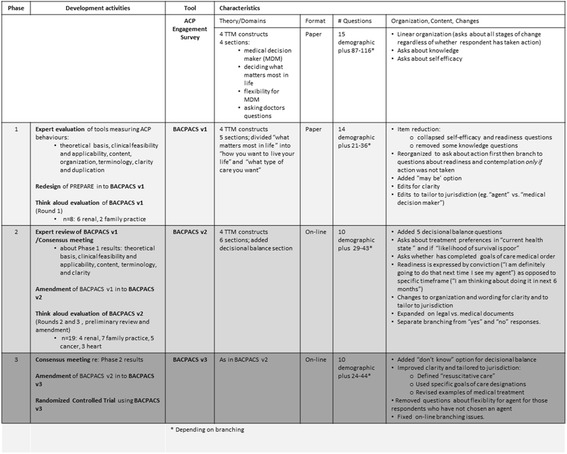
BACPACS Development and Validation Process

There were three phases to the development and refinement of the BACPACS from the ACP Engagement Survey to establish content validity and response process evidence. Figure 1 describes these phases.

### Phase 1

The first phase consisted of expert review of the content in existing tools measuring ACP behaviors, which led to the redesign of the ACP Engagement Survey into the first version of the BACPACS. During this phase items were reduced, questions were reorganized and response options were added. Cognitive interviewing [13] or pretesting with field notes (also known as verbal protocols and think-aloud interviewing) with eight patients took place.

Conversation analysis informed item reduction and survey redesign in this initial phase [14–17]. Conversation analysis is concerned with the structure, process and organization of interaction. This involved 1) sequence organization of the questions to ensure coherent, orderly and meaningful successions or ‘sequences’ of actions and 2) question design to optimize the functionality of the new BACPACS [14, 16].

### Phase 2

The second phase comprised further cognitive, think aloud interviewing [13] and expert review by the study team. The purpose of this cognitive interviewing was to apply cognitive theory to aid in the understanding of how respondents comprehended and interpreted questions in order to identify questions that elicited response error [18]. The process involved analysis of respondents’ verbal reports while completing BACPACS [19, 20]. In contrast to using verbal probes to ask patients about specific aspects of each question, the think aloud method allows the interviewer to listen and record the verbal think-aloud stream while noting the processes the participant uses in arriving at a response.

Adult patients were recruited from outpatient kidney, cancer and heart function clinics as well as family practice clinics in Edmonton, Alberta, Canada (metropolitan city, population 1 million). For logistical reasons, only English-speaking patients who were able to consent to participation without a proxy to speak on their behalf were eligible to participate. We selected participants 50 years of age and older due to the increased prevalence of chronic illness in this population and a greater relevance for ACP. We wished to validate the BACPACS in the population for which it is intended for use. Patients less than 50 years of age were excluded as age has a strong influence on how people respond to surveys.^1^

Participants were recruited to allow for the various branching options in the BACPACS (e.g. having an agent vs. not having an agent). Patient participants met with the study interviewer to determine eligibility and obtain informed consent. During the interview, the interviewer prepared the BACPACS, hosted by Research Electronic Data Capture (REDCap) [21] hosted by the Women & Children’s Health Research Institute at the University of Alberta. An iPad was used to collect participants' responses. Patients were asked to read and respond to each question aloud, and to document their response electronically. Interviews were audio recorded and listened to by the interviewer and two additional members of the research team who answered the following for each question: *1) Can the participant interpret the question? (Yes/No) If no, what seems to be the problem? 2) Can the participant answer the question with the available answer options? (Yes/No). If no, what seems to be the problem?* For each of the questions, the researchers were asked to provide evidence (or lack thereof) that the participant interpreted the question and could answer the questions on the BACPACS with the available answer options.

The qualitative research method of constant comparison [22] was used to analyze each interview. During this inductive process, themes and exceptions were identified to draw new meaning from the data. Upon review of each round of interviews, a research assistant outside of the research team summarized all of the responses for each survey question. The interviewers then discussed any discrepancies that arose. Proposed changes to the BACPACS questions were brought to the full study team to develop consensus on how best to refine the questions. Interview rounds continued until saturation had been reached, i.e., there were no further concerns by either participants or the researchers with the BACPACS questions.

### Phase 3

This phase consisted of a consensus meeting to review phase 2 results. Additional file 2 shows the final BACPACS tool for use.

## Results

### Establishing evidence for content validity

Figure 1 indicates the steps that were taken to develop the BACPACS and corresponding changes. Through key content expert review and conversation analysis, our structural and sequence organizational modifications reduced the questions asked from 116 in the ACP Engagement Survey to a maximum of 38 and a minimum of 24 questions in the BACPACS, depending on the branching sequence.^2^ Consolidation of the domains of self-efficacy and readiness also took place since patients were unable to differentiate between them. The decision for consolidation took place at the consensus meeting when all key content experts (palliative care physicians and palliative care researchers) reviewed the patient data and found responses to self-efficacy and readiness were answered in the same manner since patients did not understand the difference between the two concepts. Furthermore, patients reported during the interviews that they could not differentiate between self-efficacy and readiness.

The grouping of questions in each section of the original ACP Engagement Survey on the other hand, are sequentially organized with the assumption that respondents have not engaged in any component of ACP. Respondents are asked in all sections about their [1] thoughts [2] confidence (i.e., self-efficacy), [3] readiness, and [4] actions taken. The ACP Engagement Survey does not permit a respondent to skip irrelevant questions. If a respondent had not thought about an element of the ACP process, they are still asked to answer questions about confidence, readiness and actions. Likewise, respondents who have taken action must answer a series of questions regarding earlier steps in the process such as whether they had thought about the action and their readiness to take action before they are able to declare their action. This sequential organization creates a high, redundant response burden.

These sequence organizational issues were addressed in the BACPACS in two ways. First, a top-down approach was used in that the survey inquires about the action first and then works backwards towards patient perceptions if prior ACP actions have not been done. Second, respondents who indicate a higher-level involvement in a behavior are not asked about lower levels of engagement. This allows respondents to indicate where they are in the ACP process without redundant questions. While this can be done with paper-based surveys, electronic platforms (REDCap in our case) are particularly useful to direct patients to the next relevant question [21].

Question design for the BACPACS was informed primarily by Heritage’s (2006) question design for healthcare encounters [16, 17]. Attention was given to [1] the polarization of the questions; and [2] the singularity of question structure. Questions containing the words ‘any,’ ‘ever,’ ‘at all,’ or ‘not’ are considered negatively polarized because they are designed to expect a negative response (e.g., ‘no,’ ‘never’) and discourage elaboration. In contrast, questions that contain the word ‘some’ are considered positively polarized as they are designed to expect an affirmative or confirmative response (e.g., ‘yes’ if appropriate) and encourage elaboration. Should someone answer ‘no’ to a positively polarized question, there can be a tendency to provide justification for answering in contrast to the design of the question. Many of the questions in the ACP Engagement Survey are negatively polarized. Questions were thus redesigned to be positively polarized to increase the likelihood that respondents will seek an answer more fitting of their situation rather than gravitating toward a ‘no’ response.

A related question design issue is the use of double-barreled questions i.e., questions that touch on more than one issue but only allow for one answer. A common phrasing for questions in the ACP engagement survey was “whether or not…” something has occurred, which creates confusion and dysfunction. The BACPACS contains singular, positively polarized questions only. Items were then remapped to theoretical constructs to ensure no gaps existed in the theoretical framework. Initial item reduction was done concurrently by two research team members followed by presentation to and consensus development with all study investigators.

### Establishing evidence for response process validity

Three rounds of data collection led to the recruitment of 32 participants and all possible branching was tested throughout the administration of BACPACS (i.e. involving both people with and without an agent). Five participants were excluded from the study: one had a concurrent treatment complication, one where data recording failed, and three whose ages were less than 50 years.

There were four areas in which the BACPACS questions required further refinement. These areas related to ACP constructs, response options, as well as terminology and instructions. The BACPACS tool has a Flesch-Kincaid grade reading level of grade 8. What follows are elaborations of these themes and the changes made to the survey supported by the think aloud interviews.

#### ACP construct

Cronbach and Meehl (1955) define a construct as “a concept for which there is no single observable referent, which cannot be directly observed, and for which there exists multiple referents” [23]. Most patients had heard of legal documents, such as an advance directive. However, most participants were unaware of ACP as a range of behaviors focused predominantly on conversations and most had not thought about talking to a healthcare provider. During cognitive interviewing, we were able to determine the ways in which patients constructed the concept of ACP, and how best to describe ACP to ensure patients understood it. For example, patients queried whether they would need a lawyer for such planning and whether this would be valid within the local healthcare system. These queries were addressed during the interviews.

#### Response options

**Fig. 2:**
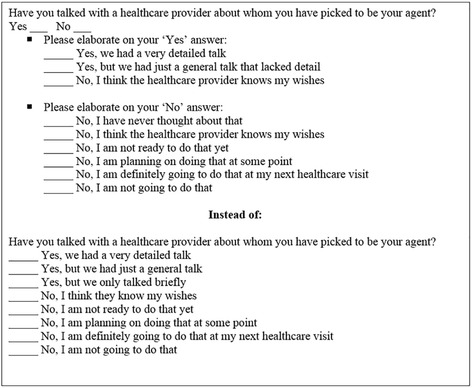
Example of Branching Yes/No questions

Patients expressed difficulty responding to questions that combined Yes and No response options coupled with explanatory detail (see Fig. 2). We incorporated branching logic to these questions, i.e. patients would first answer the root question with ‘Yes’ or ‘No’ and then would see only the corresponding response elaborations, thereby decreasing cognitive load.

#### Terminology and instructions

**Fig. 3:**
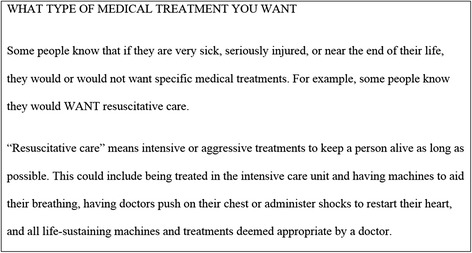
Example of Instruction Clarification

The cognitive interviewing identified healthcare terms, such as “resuscitation” that were poorly understood by participants. Instructions were therefore refined that included explicit definitions (see Fig. 3 for an example).

**Fig. 4:**
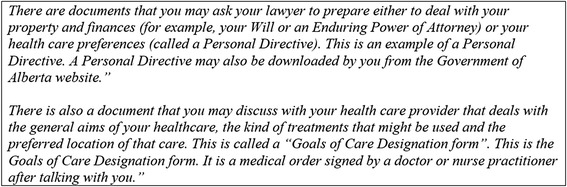
Example of Explaining Terminology Related to ACP

Participants were also confused about the various ACP documents that could be completed during ACP. Most participants had not heard of the local terminology of the medical order that communicates ACP preferences in Alberta regarding medical interventions and locations of care called a Goals of Care Designation (GCD) [24]. The questions on the BACPACS v1 and v2 (Fig. 1) did not include definitions. In BACPACS v3, the concepts of a living will, enduring power of attorney and personal directive were differentiated from the GCD and definitions were made explicit in the instructions prior to completing the relevant questions (see Fig. 4 for an example).

## Discussion

This study provided evidence for content and response process validity for a new tool used to measure patient engagement with ACP called the Behaviours in Advance Care Planning and ACtions Survey (BACPACS). The highly systematic approach to the development and validation of this tool ensures the tool is not only based on a solid theoretical framework that approaches ACP as a range of behaviors, but ensures its usability in routine clinical encounters of chronically ill patients. These patients often have poor healthcare literacy, including knowledge of ACP, are often frail, fatigue easily, and may have unrecognized cognitive impairment. All these factors are relevant when designing tools to assess interventions aimed at changing their health behaviors.

Previous tools, despite having a common theoretical framework, namely the transtheoretical model of health behavior change (see Additional file 1) have a high participant burden and high cognitive load, reducing their utility in clinical settings of older, more vulnerable patients. Recent attempts have been made to shorten the original tool by Sudore et al. [7] with the use of factor analysis. However, the use of think aloud methods were not applied and it is unclear which of the new versions warrants use as there are 55, 34, 15, 9 and 4-item versions [25]. The BACPACS is based on the TTM theory and the two qualitative methods of conversation analysis and think aloud. This resulted in a shortened, restructured and valid tool with respect to content and response process validity evidence.

There are several strengths to this study. First, it involved patients alongside content and design experts in the development of this patient engagement assessment tool. These were older, chronically ill patients who are often under-represented in research and are particularly relevant for engagement in ACP. Patients came from several disease populations within community and hospital-based settings. We also used technology in the form of an on-line version to improve the ease of administration and reduce respondent burden.

The study results highlight the need for researchers and other key stakeholders to re-visit the existing frameworks of patient engagement in ACP to ensure the constructs that are intended to be measured are actually being measured. ACP is a complex health behavior with several components and our approach to measurement and evaluation must evolve as our understanding grows. There may be assumptions that patients are aware of ACP terminology and are well informed about the ways to document. This study challenges those assumptions. Compared to previous ACP engagement surveys, the BACPACS has fewer items, contains branching logic, is feasible for and has evidence for validity for use in the population for which it is intended, and is adaptable for use in local contexts. For example, in our local area we incorporated the GCD. Other healthcare systems can adapt the BACPACS similarly.

BACPACS shows evidence for content and response process validity for measuring patient engagement of chronically ill, elderly patients in ACP. To date, no other study has conducted a thorough analysis applying conversation analysis for item reduction and the cognitive testing for quality assurance in developing a measure of patient engagement in ACP.

This study was limited to English speaking adults 50 years and older. Further work is needed in translating the survey and using it in different age groups, ethnic backgrounds and perhaps with other illnesses. BACPACS is currently being used to collect data from 240 patients in a randomized control trial on the consequences of viewing a health services video created to enhance patient understanding and engagement in ACP. These data will be used to more fully determine the psychometric properties of BACPACS such as responsiveness to change, effect sizes, relationships with other variables and to determine how best to score survey responses in detecting behavior changes over time.

## Conclusions

Conversation analysis, content expert review and think aloud cognitive interviewing were useful in refining the new survey instrument entitled BACPACS. We found evidence for both content and response process validity for this new tool. There is a need for valid ACP survey tools to understand where people are in this process to guide health policy and clinical practice. The BACPACS offers an excellent starting point.

## Additional files

Additional file 1: Consensus of a theoretical framework and the constructs to be included for ACP. (PDF 35 kb)

Additional file 2: Behaviours in Advance Care Planning and Actions Survey (BACPACS). (PDF 558 kb)

## Abbreviations

ACP: Advance care planning
AERA: American Educational Research Association
APA: American Psychological Association
BACPACS: Behaviours in Advance Care Planning and ACtions Survey
GCD: Goals of Care Designation
NCME: National Council on Measurement in Education
REDCap: Research Electronic Data Capture
TTM: Trans-theoretical model of behavior change
UA HREB: University of Alberta Health Research Ethics Board

## Footnotes

1 Round 1 included *n* = 8 patients of which 6 were from renal dialysis and 2 from family clinics. For rounds 2 and 3, there were 4 from renal dialysis units (1 in round 2, 3 in round 3), 7 from family clinics (2 in round 2, 5 in round 3), 5 from cancer outpatient clinics (2 in round 2, 3 in round 3) and 3 from heart failure outpatient clinics (3 in round 3). We also had 5 patients that we describe in the paper as excluded (3 that were under the age of 50, one cancer patient that had a complication and one where the recording failed). The total is 32: 8 (round 1) *n* = 19 (round 2 and 3) *n* = 5 excluded.

2 The initial revised version of the ACP survey contains branching logic. That is, appearance of subsequent questions is dependent upon the answer of root questions. Consequently, respondents will be asked different follow up questions depending on whether they answered ‘yes’ or ‘no’ to the root question. In most cases, answering ‘yes’ resulted in more follow up questions than answering ‘no.’
