# Supplementary material for: Behaviors in Advance Care Planning and ACtions Survey (BACPACS): development and validation part 1
Source: BMC Palliat Care. 2017 Nov 22;16:56. doi: 10.1186/s12904-017-0236-6 (PMC5700663; doi:10.1186/s12904-017-0236-6)
Supplement: Supplementary file 1 — Consensus of a theoretical framework and the constructs to be included for ACP. (PDF 35 kb) [file 12904_2017_236_MOESM1_ESM.pdf]

Consensus of a theoretical framework and the constructs to be included for ACP

A recent scoping review[8] of the literature conducted by several of the authors from the current study identified the trans-theoretical model (TTM) or “Stages of Change” model as the theoretical framework most commonly used to understand and assess people’s readiness to engage in various health behaviors among a diverse range of health conditions. Three of the four tools reflect the work of Sudore and Fried and are based on the TTM model.

The TTM model has evolved over time but currently includes 4 constructs: 1) stages of change, 2) decisional balance, 3) processes of change, and 4) self-efficacy. Stages of change involve pre-contemplation (no intention to change in the near future), contemplation (thinking about changing in the near future), preparation (commitment to changing the behavior soon), action (a recent change in behavior), and maintenance (ongoing behavior change). Decisional balance takes into account an individual’s weighing of the pros and cons of changing their behaviors. It assesses patient’s attitudes about common barriers to and facilitators of behavior change, which may be influenced by factors such as religious beliefs and medical misconceptions. Process of change measures overt and covert activities or strategies that people use to progress through the stages of change. It should be noted that self-efficacy as a construct is often embedded in the other three constructs and patients may be unable to distinguish between them, therefore creating redundancy. The theoretical constructs and ACP behaviours incorporated into existing ACP readiness assessment tools are summarized in Table 1.

Table 1: Tools for Measuring ACP Behaviors

Tool	Theoretical framework	Description	Evidence for validity	Strengths	Limitations
PREPARE Survey[9]<br><br>(Sudore et al.,2013)	TTM, Social Cognitive Theory, Interpersonal communication competence model and Behavior Change Theory	Measures all 4 TTM constructs, including patient knowledge (health literacy), self-efficacy, contemplation and readiness	Pilot tested (n=50) and evaluated for internal consistency and test-retest reliability.	Highly comprehensive	There are 116 questions, which limits clinical usability.<br><br>No branching logic
Focus Groups[3]<br><br>Fried et al., (2009)	TTM	Measures 1 of the 3 TTM constructs: stages of change	None provided	Short	Not a tool: qualitative focus groups
Scales to measure Decisional Balance, Medical and Religious Beliefs, and Processes of Change<br><br>Fried et al., (2012)	TTM	Measures 3 of 4 TTM constructs (stages of change, decisional balance and process of change) using 12, 7 and 15 (total of 34) items respectively.	Good to excellent internal consistency (Cronbach α .76–.93)	Covers most TTM constructs<br><br>Relatively short	Does not measure self-efficacy<br><br>Difficult for participants to understand items<br><br>No logic branching

As shown in Table 1, Fried et al., have applied the TTM to ACP, measuring not only stages of readiness but also decisional balance and process of change. However, Sudore's (2013) PREPARE survey builds further on knowledge of behavior change, adding an important aspect

of behavior change, namely self-efficacy.[9] Fried et al., (2009, 2012) found Sudore had focused on stages of change measures and had not developed measures for the other TTM constructs of decisional balance and process of change. Yet, Sudore (2013) clearly includes some processes of change and attitudes (relating to quality of life). Given the above and that Sudore's tool measures self-efficacy whereas Fried's does not, we selected the PREPARE survey as a better starting point for the development of the new ACP engagement measure, BACPACS.
