# Supplementary material for: Behaviors in Advance Care Planning and ACtions Survey (BACPACS): development and validation part 1
Source: BMC Palliat Care. 2017 Nov 22;16:56. doi: 10.1186/s12904-017-0236-6 (PMC5700663; doi:10.1186/s12904-017-0236-6)
Supplement: Supplementary file 2 — Behaviours in Advance Care Planning and Actions Survey (BACPACS). (PDF 558 kb) [file 12904_2017_236_MOESM2_ESM.pdf]

Effectiveness and Economic Impact of Advance Care Planning and Goals of Care Designations Discussions:
A Randomized Controlled Trial

Behaviours in Advance Care Planning and Actions Survey (BACPACS)

**Subject Study ID:** ☐☐☐-☐-☐☐☐-☐☐☐<br>Site number-Centre-Enrolment number-Participant's initials	**Visit Date:** __ __/ __ __ __/ 2 0_ __ __<br>d d m m y y y y<br>**Start time:** ____________________

Accidents happen. Illness happens. Advance care planning helps to honour patient preferences, wishes and goals if illness or injury prevents someone from speaking for him or herself. We would like to learn about your experiences with making future healthcare decisions for yourself.

Demographics

1. What is your age? ___________ years

2. Are you : _____ Female _____ Male

3. What is your current marital status?

_____ Never legally married
_____ Divorced
_____ Widowed
_____ Legally married (and not separated)
_____ Common law (living as married)
_____ Separated but still legally married

RCT of ACP & GCD Discussions
Ethics ID: Pro00054953 (HREB), REB14-1151 (CHREB)
Version 2.1, Nov 16, 2015

4. What is the highest level of education you have received?

_____ Some high school or less
_____ High school diploma
_____ Some college or trade school
_____ College or trade school diploma
_____ Some university
_____ University degree
_____ Post-graduate

5. Besides English, do you speak another language on a regular basis at home?

_____ Yes _____ No

5a. You indicated that you speak another language besides English. What other language do you speak on a regular basis at home?

____

6. People sometimes identify themselves by 'race' and/or colour. Please check beside the group or groups for which you MOST identify.

_____ White
_____ Chinese
_____ Filipino
_____ Arab
_____ Korean
_____ Aboriginal (for example, First Nations, Metis, etc)
_____Black
_____ Latin American
_____West Asian (for example, Iranian, Afghan, etc)
_____ Japanese
_____ South Asian (for example, East Indian, Pakistani, Sri Lankan, etc)
_____ Southeast Asian (for example, Vietnamese, Cambodian, Malaysian, Laotian, etc)
_____ Other (please specify): ____

7. How important is religion or spirituality in your life?

_____ Extremely important

_____ Very important

_____ Somewhat important

_____ Not very important

_____ Not important

8. Do you identify with a religious or spiritual group or practice?

_____ Yes _____ No _____ Maybe

8a. If you indicated that you identify with a religion, what is your religion?

_____ Roman Catholic

_____ Anglican

_____ Lutheran

_____ Presbyterian

_____ Jewish

_____ Hindu

_____ Greek Orthodox

_____ United Church

_____ Baptist

_____Muslim

_____ Pentecostal

_____ Buddhist

_____ Sikh

_____ Protestant

_____ Other (please specify):

____

9. Do you live alone?

_____ Yes _____ No

10. Do you have a healthcare provider (for example, a nurse, doctor, nursing aid) come to your residence to provide you with medical care?

______ Yes ______ No

WHAT IS IMPORTANT TO YOU IN YOUR LIFE

Sometimes people are faced with difficult choices when they are seriously hurt or very sick. Some people have very specific preferences and wishes when it comes to the type of healthcare that is right for them. These preferences and wishes may be based on past experiences, family and/or personal values, and/or spiritual beliefs. What is important to you in your life may be different or the same as other people you know. What matters is that you receive the type of healthcare that is right for you.

Please rate how important each of the following items is to you. There are no right or wrong answers to the following questions. Please answer the questions as honestly as you can.

11. When I am ill or injured I want to learn about the type of healthcare that is available to me.

_____ Extremely important
_____ Very important
_____ Moderately important
_____ Somewhat important
_____ Not important
_____ Don't know

12. I want to decide ahead of time what type of health situations are and are not acceptable to me (for example, being in a coma, not being able to move around or talk with my family/friends).

_____ Extremely important

_____ Very important

_____ Moderately important

_____ Somewhat important

_____ Not important

_____ Don't know

13. I want to know the truth about my health even if it is not good news.

_____ Extremely important

_____ Very important

_____ Moderately important

_____ Somewhat important

_____ Not important

_____ Don't know

14. I want to pick someone to speak for me in healthcare situations, such as a family member or close friend, if I am not able to speak for myself (for example, if I am unconscious or I had a stroke and my speech was impaired).

_____ Extremely important

_____ Very important

_____ Moderately important

_____ Somewhat important

_____ Not important

_____ Don’t know

There are documents that you may ask your lawyer to prepare, either to deal with your property and finances (for example, a Will or an Enduring Power of Attorney) or your healthcare preferences (called a Personal Directive). This is an example of a Personal Directive. A Personal Directive may also be downloaded by you from the Government of Alberta website.

There is also a document that you may discuss with your health care provider that deals with the general aims of your healthcare, the kind of treatments that might be used and the preferred location of that care. This is called a "Goals of Care Designation form". This is the Goals of Care Designation form. It is a medical order signed by a doctor or nurse practitioner after talking with you.

15. I want to have some type of documents (for example, goals of care designation medical order form, personal directive, living will) that tell healthcare professionals the type of medical care that is acceptable to me if I cannot speak for myself.

_____ Extremely important

_____ Very important

_____ Moderately important

_____ Somewhat important

_____ Not important

_____ Don't know

AN AGENT

An agent is someone you trust who will tell healthcare providers what medical actions are acceptable to you should you be unable to speak for yourself. You can have more than one agent if you choose. An agent can be anyone you chose, such as a family member or close friend.

Agents can also be called alternative decision makers or surrogate decision makers.

The following questions relate to your wishes regarding an agent. There are no right or wrong answers to the following questions. Please answer the questions as honestly as you can.

16. This question asks about a decision you may have already made in your own mind, even if you haven't talked with anyone about it yet. Have you already decided who you want your agent or agents to be?

    _____ Yes   _____ No

If you answered 'No' to Question 16, please go to Question 17 (page 14)

If you answered 'Yes' to Question 16, please answer questions on the following page:

16a. What relationship is this person (are these people) to you?

Please click by all that apply.

_____ Spouse/partner	_____ Daughter/son
_____ Mother/father	_____ Sister/brother
_____ Grandmother/grandfather	_____ Niece/nephew
_____ Aunt/uncle	_____ Friend
_____ Family/Group	_____ Other (please specify):

____

16b. Have you asked this person to be your agent?

_____ Yes _____ No

Please elaborate on your 'Yes' answer:

_____ Yes, we had detailed talks

_____ Yes, but we had just a general talk that lacked detail

_____ No, I think this person knows my wishes

Please elaborate on your 'No' answer

_____ No, I have never thought about that

_____ No, I think this person knows my wishes

_____ No, I am not ready to do that yet

_____ No, I am planning on doing that at some point

_____ No, I am definitely going to do that next time I see this person

_____ No, I am not going to do that

16c. Have you signed some type of document (for example, personal directive, living will) naming the person you selected to be your agent?

_____ Yes _____ No _____ Don't know

If you answered 'Yes' to Question 16c, please proceed to Question 16d.

If you answered 'No' or 'Don't know' to Question 16c, please answer the next question:

16cc. How ready are you to sign a document putting in writing who you would want to be your agent (that is, completing a personal directive)?

_____ I have never thought about it
_____ I have thought about it, but I am not ready to do it
_____ I have thought about it and I am definitely not going to do it
_____ I am thinking about doing it
_____ I am definitely planning to do it

16d. Have you talked with a healthcare provider about whom you have picked to be your agent?

_____ Yes _____ No

Please elaborate on your ‘Yes’ answer:

_____ Yes, we had detailed talks

_____ Yes, but we had just a general talk that lacked detail

_____ No, I think the healthcare provider knows my wishes

Please elaborate on your ‘No’ answer:

_____ No, I have never thought about that

_____ No, I think the healthcare provider knows my wishes

_____ No, I am not ready to do that yet

_____ No, I am planning on doing that at some point

_____ No, I am definitely going to do that at my next healthcare visit

_____ No, I am not going to do that

If you answered “No” to Question 16d, please go to Question 16f (page 13)

If you answered “Yes” to Question 16d, please answer the next question:

16e. Who did you talk with about your agent? (Please click all that apply)

_____ Doctor

_____ Nurse Practitioner

_____ Nurse

_____ Home Care Worker

_____ Social Worker

_____ Long Term Care Facility Staff

_____ Pharmacist

_____ Occupational Therapist

_____ Physiotherapist

_____ Other (please specify):

____

16f. Have you talked with other family and/or friends about who you have picked to be your agent?

_____ Yes _____ No

Please elaborate on your 'Yes' answer:

_____ Yes, we had detailed talks

_____ Yes, but we had just a general talk that lacked detail

_____ No, I think my other family/friends know who I want to be my agent

Please elaborate on your 'No' answer:

_____ No, I have never thought about that

_____ No, I think my other family/friends know who I want to be my agent

_____ No, I am not ready to do that yet

_____ No, I am planning on doing that at some point

_____ No, I am definitely going to do that next time I see my family/friends

_____ No, I am not going to do that

After Question 16f, please proceed to the <u>next section</u> ("TALKING ABOUT WHAT IS IMPORTANT IN YOUR LIFE") (page 17)

17. If you answered "No" to Question 16, please elaborate on your 'No' answer:

______ No, I have never thought about that

______ No, I am not ready to do that yet

______ No, I am planning on doing that at some point

______ No, I am definitely going to do that

______ No, I am not going to do that

17a. Have you talked with your health care provider about who you might want to be your agent?

______ Yes ______ No

Please elaborate on your 'Yes' answer:

______ Yes, we had detailed talks

______ Yes, but we had just general talks that lacked detail

______ No, I think the health care provider knows my wishes

Please elaborate on your 'No' answer:

______ No, I have never thought about that

______ No, I think the health care provider know who I want to be my agent

______ No, I am not ready to do that yet

______ No, I am planning on doing that at some point

______ No, I am definitely going to do that at my next health care visit

______ No, I am not going to do that

If you answered "No" to Question 17a, please go to Question 18

If you answered "Yes" to Question 17a, please answer the next question:

17aa. Who did you talk with about who you might want to be your agent? (Please check all that apply)

_____ Doctor
_____ Nurse Practitioner
_____ Nurse
_____ Home Care Worker
_____ Social Worker
_____ Long Term Care Facility Staff
_____ Pharmacist
_____ Occupational Therapist
_____ Physiotherapist
_____ Other (please specify):

____

18. Have you talked with your family and/or friends about who you might want to be your agent?

_____ Yes _____ No

Please elaborate on your ‘Yes’ answer:

______ Yes, we had detailed talks

______ Yes, but we had just general talks that lacked detail

______ No, I think my wishes are known

Please elaborate on your ‘No’ answer:

______ No, I have never thought about that

______ No, I think my family/friends know who I want to be my agent

______ No, I am not ready to do that yet

______ No, I am planning on doing that at some point

______ No, I am definitely going to do that next time I see my family/friends

______ No, I am not going to do that

TALKING ABOUT WHAT IS IMPORTANT TO YOU IN YOUR LIFE

Sometimes people are faced with difficult choices when they are seriously hurt or very sick. Some people want to live no matter what the situation (for example, being in a coma, not being able to get out of bed). Other people believe that their life would not be worth living in some situations.

There are no right or wrong answers to the following questions. Please answer the questions as honestly as you can.

If you have NOT DECIDED WHO YOU WANT YOUR AGENT TO BE, please proceed to the Question 20 (page 21)

If you HAVE DECIDED WHO YOU WANT YOUR AGENT TO BE, please answer the following questions:

19. Have you told your agent about the type of healthcare you would want if you could not speak for yourself?

_____ Yes   _____ No

Please elaborate on your 'Yes' answer:

_____ Yes, we had detailed talks

_____ Yes, but we had just a general talk that lacked detail

_____ No, I think my agent knows my wishes

Please elaborate on your 'No' answer:

_____No, I have never thought about that

_____ No, I think my agent knows my wishes

_____ No, I am not ready to do that yet

_____ No, I am planning on doing that at some point

_____ No, I am definitely going to do that next time I see my agent

_____ No, I am not going to do that

19a. Have you told a healthcare provider about the type of healthcare you would want if you could not speak for yourself?

_____ Yes _____ No

Please elaborate on your 'Yes' answer:

______ Yes, we had detailed talks

______ Yes, but we had just a general talk that lacked detail

______ No, I think the healthcare provider knows my wishes

Please elaborate on your 'No' answer:

______ No, I have never thought about that

______ No, I think the healthcare provider knows my wishes

______ No, I am not ready to do that yet

______ No, I am planning on doing that at some point

______ No, I am definitely going to do that at my next healthcare visit

______ No, I am not going to do that

If you answered 'Yes' to Question 19a, please answer Question 19aa

If you answered 'No' to Question 19a, please proceed to Question 19b (page 20)

19aa. Who did you talk with about your healthcare wishes? **(Please check all that apply)**

______ Doctor

______ Nurse Practitioner

______ Nurse

______ Home Care Worker

______ Social Worker

______ Long Term Care Facility Staff

______ Pharmacist

______ Occupational Therapist

______ Physiotherapist

______ Other (please specify):

19b. Have you told your family and/or friends (other than your agent) about the type of healthcare you would want if you could not speak for yourself?

_____ Yes   _____ No

Please elaborate on your 'Yes' answer:

_____ Yes, we had detailed talks

_____ Yes, but we had just general talks that lacked detail

_____ No, I think my other family/friends know my wishes

Please elaborate on your 'No' answer:

_____ No, I have never thought about that

_____ No, I think my other family/friends know my wishes

_____ No, I am not ready to do that yet

_____ No, I am planning on doing that at some point

_____ No, I am definitely going to do that next time I see them

_____ No, I am not going to talk to my other family/friends about this

19c. Do you have a Personal Directive telling people the type of healthcare you want if you could not speak for yourself? (In some jurisdictions, this may have another name such as Advance Directive, Living Will).

_____ Yes   _____ No   _____ Don't know

If you answered 'Yes' to Question 19c, please proceed to the next section ("WHAT TYPE OF MEDICAL TREATMENT YOU WANT" (page 25)

If you answered 'No' or 'Don't know' to Question 19c, please answer Question 19d.

19d. How ready are you to document the type of healthcare you want in a Personal Directive if you could not speak for yourself?

_____ I have never thought about it

_____ I have thought about it, but I am not ready to do it

_____ I have thought about it and I am definitely not going to do it

_____ I am thinking about doing it

_____ I am definitely planning to do it

If you HAVE DECIDED WHO YOU WANT TO BE YOUR AGENT, please proceed to the next section ("WHAT TYPE OF MEDICAL TREATMENT YOU WANT") (page 25)

If you have NOT DECIDED WHO YOU WANT YOUR AGENT TO BE, please answer the following questions:

20. Have you told a healthcare provider about the type of healthcare you would want if you could not speak for yourself?

_____ Yes _____ No

Please elaborate on your 'Yes' answer:

_____ Yes, we had detailed talks

_____ Yes, but we had just a general talk that lacked detail

_____ No, I think the healthcare provider knows my wishes

Please elaborate on your 'No' answer:

_____ No, I have never thought about that

_____ No, I think the healthcare provider knows my wishes

_____ No, I am not ready to do that yet

_____ No, I am planning on doing that at some point

_____ No, I am definitely going to do that at my next healthcare visit

_____ No, I am not going to do that

If you answered 'Yes' to Question 20, please answer the next question.

If you answered 'No' to Question 20, please proceed to Question 20b (page 23).

20a. Who did you talk with about your healthcare wishes? **(Please check all that apply)**

_____ Doctor

_____ Nurse Practitioner

_____ Nurse

_____ Home Care Worker

_____ Long Term Care Facility Staff

_____ Pharmacist

_____ Occupational Therapist

_____ Physiotherapist

_____ Social Worker _____ Other (please specify):

____

20b. Have you told your family and/or friends about the type of healthcare you would want if you could not speak for yourself?

_____ Yes _____ No

Please elaborate on your 'Yes' answer:

_____ Yes, we had detailed talks

_____ Yes, but we had just general talks that lacked detail

_____ No, I think my family/friends know my wishes

Please elaborate on your 'No' answer:

_____ No, I have never thought about that

_____ No, I think my family/friends know my wishes

_____ No, I am not ready to do that yet

_____ No, I am planning on doing that at some point

_____ No, I am definitely going to do that next time I see them

_____ No, I am not going to talk to my family/friends about this

20c. Do you have a Personal Directive telling people the type of healthcare you want if you could not speak for yourself? (In some jurisdictions, this may have another name such as Advance Directive, Living Will).

_____ Yes _____ No _____ Don't know

If you answered 'Yes' to Question 20d, please proceed to the next section ("WHAT TYPE OF MEDICAL TREATMENT YOU WANT") (page 25).

If you answered 'No' or 'Don't know' to Question 20c, please answer Question 20d.

20d. How ready are you to document the type of healthcare you want in a Personal Directive if you could not speak for yourself?

_____ I have never thought about it

_____ I have thought about it, but I am not ready to do it

_____ I have thought about it and I am definitely not going to do it

_____ I am thinking about doing it

_____ I am definitely planning to do it

UNIVERSITY OF ALBERTA | UNIVERSITY OF CALGARY | Alberta Health Services | Covenant Health | ACP CRIO

WHAT TYPE OF MEDICAL TREATMENT YOU WANT

Some people know that if they are very sick, seriously injured, or near the end of their life, they would or would not want specific medical treatments. For example, some people know they would WANT resuscitative care.

"Resuscitative care" means intensive or aggressive treatments to keep a person alive as long as possible. This includes being treated in the intensive care unit and may include having machines to aid their breathing, having doctors push on their chest or administer shocks to restart their heart, and all life-sustaining machines and treatments deemed appropriate by a doctor. Other people know they would NOT WANT resuscitative care.

Generally speaking, the Goals of Care Designation is used by healthcare providers to describe the general aims of your healthcare, the kinds of treatments that might be used and the preferred location of that care.

A Goals of Care Designation is a medical order signed by a doctor or nurse practitioner after talking with you. A Goals of Care Designation can be changed at any time.

The type of healthcare you receive is often referred to as a 'Goal of Care'. There are no right or wrong answers to the following questions. Please answer as honestly as you can.

21. Based on your CURRENT HEALTH STATE, which goals of care designation is important to you?

_____ Resuscitative care: I want my life prolonged or preserved using any medical or surgical means including being treated in the intensive care unit and, if needed, having machines to aid my breathing, having doctors push on my chest or administer shocks to restart my heart, and all life-sustaining machines and treatments deemed appropriate by a doctor.

_____ Medical care: I want medical tests and interventions to be used to cure or manage an illness as well as possible, but I don't want resuscitation.

_____ Comfort care: I want to be provided with comfort care to ease my symptoms without trying to control the underlying illness.

_____ I am not ready to answer this question

22. When your likelihood of SURVIVAL IS POOR, what goals of care designation would be important to you? **(Please see more response options on following page)**

_____ Resuscitative care: I want my life prolonged or preserved using any medical or surgical means including being treated in the intensive care unit and, if needed, having machines to aid my breathing, having doctors push on my chest or administer shocks to restart my heart, and all life-sustaining machines and treatments deemed appropriate by a doctor.

_____ Medical care: I want medical tests and interventions to be used to cure or manage an illness as well as possible, but I don't want resuscitation.

_____ Comfort care: I want to be provided with comfort care to ease my symptoms without trying to control the underlying illness.

_____ I am not ready to answer this question

23. Do you already have a completed goals of care designation order form?

_____ Yes _____ No _____ Don’t know

If you answered ‘Yes’ to Question 23, please proceed to Question 23a.

If you answered ‘No’ or ‘Don’t know’ to Question 23, please answer Question 23c (page 28)

23a. To the best of your recollection, what is the designation on your goals of care designation order form?

____R ____M ____C

____R1 ____R2 ____R3

____M1 ____M2

____C1 ____C2 ____C3

____ Don’t know

23b. To the best of your recollection, when was this goals of care designation form completed?

_____ ≤ 3 months

_____ 3-12 months

_____> 12 months

If you have completed goals of care designation form, please go to next section ("FLEXIBILITY FOR AN AGENT") (page 29)

23c. How ready are you to have your goals of care designation put in writing?

_____ I have never thought about it

_____ I have thought about it, but I am not ready to do it

_____ I have thought about it and I will do it if my health state changes or if I'm hospitalized for any reason

_____ I have thought about it and I am definitely not going to do it

_____ I am thinking about it

_____ I am definitely planning to do it

FLEXIBILITY FOR THE AGENT

Flexibility for the Agent means that if you are unable to speak for yourself, your agent has your permission to change your prior medical decisions if a doctor or your agent believe it is the best for you at the time. Giving flexibility to your agent is your choice.

There are no right or wrong answers to the following questions. Please answer the questions as honestly as you can.

If you have NOT DECIDED WHO YOU WANT TO BE YOUR AGENT, please go to next section ("ASKING YOUR DOCTOR QUESTIONS") (page 35)

If you HAVE DECIDED AN AGENT, please answer the following questions:

24 . Thinking about the flexibility you would want your agent to have, please pick the answer that is most appropriate for you.

_____ I would want my agent to be able to change any of my medical decisions if the doctors think it is best for me at that time

_____ I would want my agent to be able to change some of my medical decisions. But some decisions I NEVER want changed, even if the doctors recommend it

_____ I would want my agent to follow all of my medical wishes exactly, no matter what. It is NOT OKAY to change my decisions, even if the doctors recommend it.

_____ I am not ready to answer this question

24a. Have you talked with your agent about whether or not he/she can change your prior medical decisions if a doctor and your agent believe it is best for you?

_____ Yes _____ No

Please elaborate on your 'Yes' answer:

_____ Yes, we had detailed talks

_____ Yes, but we had just a general talk that lacked detail

_____ No, I think my agent knows my wishes

Please elaborate on your 'No' answer:

_____No, I have never thought about that

_____ No, I think my agent knows my wishes

_____ No, I am not ready to do that yet

_____ No, I am planning on doing that at some point

_____ No, I am definitely going to do that next time I see my agent

_____ No, I am not going to do that

24b. Have you talked with your family and/or friends (other than your agent) about whether or not your agent can change your prior medical decisions if a doctor and your agent believe it is best for you?

_____ Yes _____ No

Please elaborate on your ‘Yes’ answer:

______ Yes, we had detailed talks

______ Yes, but we had just general talks that lacked detail

______ No, I think my other family/friends know my wishes

Please elaborate on your ‘No’ answer:

______ No, I have never thought about that

______ No, I think my other family/friends know my wishes

______ No, I am not ready to do that yet

______ No, I am planning on doing that at some point

______ No, I am definitely going to do that next time I see them

______ No, I am not going to talk to my other family/friends about this

24bb. Have you talked with a healthcare provider about whether or not your agent can change your prior medical decisions if a doctor and your agent believed it is best for you?

______ Yes ______ No

Please elaborate on your ‘Yes’ answer:

_____ Yes, we had detailed talks

_____ Yes, but we had just a general talk that lacked detail

_____ No, I think the healthcare provider knows my wishes

Please elaborate on your ‘No’ answer:

_____ No, I have never thought about that

_____ No, I think the healthcare provider knows my wishes

_____ No, I am not ready to do that yet

_____ No, I am planning on doing that at some point

_____ No, I am definitely going to do that at my next healthcare visit

_____ No, I am not going to do that

24c. Have you filled in a document (for example, personal directive, living will) that says whether or not your agent can change your prior medical decisions if a doctor and your agent believe it is best for you?

_____ Yes _____ No _____ Don’t know

If you answered ‘Yes’ to Question 24c please proceed to the next section (“ASKING A DOCTOR QUESTIONS”) (page 35)

If you answered ‘No’ or ‘Don’t know’ to Question 24c, please answer Question 24d.

UNIVERSITY OF ALBERTA UNIVERSITY OF CALGARY Alberta Health Services Covenant Health ACP CRIO

24d. How ready are you to sign a document (for example, personal directive, living will) putting in writing whether or not your agent can change your prior medical decisions if a doctor and your agent believe it is best for you?

_____ I have never thought about it

_____ I have thought about it, but I am not ready to do it

_____ I have thought about it and I am definitely not going to do it

_____ I am thinking about doing it

_____ I am definitely planning to do it

Please proceed to next section ("ASKING A DOCTOR QUESTIONS") (page 35)

ASKING A DOCTOR QUESTIONS

Doctors in Alberta are usually knowledgeable about advance care planning and goals of care designations.

Please answer the following questions about asking a doctor about the types of health decisions you may want to make.

There are no right or wrong answers to the following questions. Please answer the questions as honestly as you can.

25. Has a doctor discussed your options if you become seriously ill or are injured?

_____ Yes _____ No _____ Maybe

If you answered 'No' to Question 25, please proceed to Question 26 (page 36)

If you answered 'Yes' or 'Maybe' to Question 25, please answer the following question.

25a. What options has a doctor discussed with you if you become seriously ill or are injured? **(Please click beside all of the options a doctor has discussed with you.)**

_____ Resuscitative care: I want my life prolonged or preserved using any medical or surgical means including being treated in the intensive care unit and, if needed, having machines to aid my breathing, having doctors push on my chest or administer shocks to restart my heart, and all life-sustaining machines and treatments deemed appropriate by a doctor.

_____ Medical care: I want medical tests and interventions to be used to cure or manage an illness as well as possible, but I don't want resuscitation.

_____ Comfort care: I want to be provided with comfort care to ease my symptoms without trying to control the underlying illness.

_____ I am not ready to answer this question

26. Has a doctor told you what your quality of life would be like after having different treatments should you be injured or become ill in the future?

_____ Yes _____ No _____ Maybe

27. Do you feel comfortable talking with a doctor about the different kinds of treatments you could get should you be injured or become ill in the future?

_____ Yes, I feel very comfortable

_____ Yes, I feel mostly comfortable

_____ Yes, I feel somewhat comfortable

_____ No, I don't feel comfortable

Comments:

This concludes the survey. We greatly appreciate your help with improving this survey for future use. Thinking back on the survey, if there are some things about the survey that you think would help us to improve it, please write that below or tell the researcher.

Thank you very much for assisting us with this survey!

Have a good day!

____

____

____

____

____

Time Completed: ______________________
